# Somatic Mutation Profile in Normal Tissue Adjacent to Tumor in Colorectal Carcinoma Patients: Functional Effect on Gene Expression and Potential Implications for Precision Medicine

**DOI:** 10.3390/cancers18183034

**Published:** 2026-09-18

**Authors:** Farzana Jasmine, Daniil Vasiljevs, Armando Almazan, Habibul Ahsan, Muhammad G. Kibriya

**Affiliations:** 1 Institute for Population and Precision Health (IPPH), University of Chicago, Chicago, IL 60637, USA; farzana@uchicago.edu (F.J.); daniil.vasiljevs@bsd.uchicago.edu (D.V.); armando.almazan@bsd.uchicago.edu (A.A.); hahsan@bsd.uchicago.edu (H.A.); 2 Department of Public Health Sciences, University of Chicago, Chicago, IL 60637, USA

**Keywords:** somatic mutation, colorectal carcinoma, normal colon, precision medicine, transcriptome, pseudolaric acid A (PAA), TH9619

## Abstract

**Background:** The somatic mutation (SM) profile of normal colon tissue in colorectal carcinoma (CRC) patients is not well-known. We examined (a) SMs in resected normal colonic tissue adjacent to tumors from non-metastatic CRC patients, (b) identified potentially malignant mutations, and (c) their functional effects and utility for precision medicine. **Materials and Methods:** Surgically resected fresh paired tumor and adjacent normal colon tissues from 61 patients were used as sources of tissue DNA. Whole blood (WB) DNA was used as a germline reference. Next-generation sequencing using a custom hybridization capture panel was performed on each. Differential gene expression was assessed and stratified by mutation status in normal tissue. **Results:** Compared to WB DNA, similar numbers of SMs were detected in tumor and adjacent normal colon tissue. Of all SMs found in adjacent tissue, more than 23% of those same mutations were also detected in the matched tumor. Of the 61 patients, 57 (93.4%) had at least one potentially malignant non-synonymous SM in their normal tissue. The most frequently involved genes were *BRCA2* (67% of the patients), *KMT2C* (64%), *DHFR* (49%), *APC* (41%), *MAP3K1* (41%), *PIK3CA* (39%), *PTEN* (39%), *ARID1A* (38%), and *MSH6* (38%). The use of WB as a germline reference was important, as using only adjacent normal tissue as a reference caused false negatives for mutations shared between tumor and normal tissue. Mutation status in normal tissue was associated with differential gene expressions in matched tumors. *APC* mutations were associated with more marked down-regulation of *APC*, *SLC26A3*, *SELENBP1*, *KRT20*, and *CXCL14*, and more marked up-regulation of *MMP1*, *MMP12*, *MTHFD1L* and *MTHFD2*, the latter two suggesting the folate pathway as a potential therapeutic target. **Conclusions:** For the first time, we document a comprehensive SM profile of normal colon tissue adjacent to tumors in CRC patients and observe its functional effect on differential gene expression in matched tumor tissue to identify potential therapeutic applications in precision medicine.

## Simple Summary

Colorectal carcinoma (CRC) is one of the most common cancers worldwide. This study examined whether mutations found in adjacent normal colon tissue from CRC patients influence the gene expression patterns of the resulting tumor. Using matched tumor, normal colon, and whole blood samples from 61 CRC patients, we identified somatic mutations in normal colon tissue in several known cancer driver genes, including *APC*. We then determined whether tumors arising in patients who carried these mutations in their normal tissue had differential gene expressions compared to tumors from patients who did not. We observed that mutations in normal colon tissue were associated with distinct patterns of altered gene expression in matched tumor and demonstrated that using whole blood as a germline DNA reference is crucial to avoiding false negatives during variant calling.

## 1. Introduction

Colorectal carcinoma (CRC) is the second leading cause of cancer-related mortality globally, responsible for approximately 1.9 million new cases and ~900,000 deaths in 2022 [1]. In studies working to understand the mutational profile of CRC tumor tissue, advances in next-generation sequencing (NGS) have shown somatic mutations (SMs) in genes like *APC*, *TP53*, *KRAS*, *PIK3CA*, *FBXW7*, *SMAD4*, *TCF7L2* and *NRAS* [2]. They accumulate during the adenoma-carcinoma sequence [3] and have been shown to be useful as prognostic biomarkers and potential therapeutic targets [4,5,6].

However, most genomic studies in CRC focus on tumor tissue only. The SM profile of normal colon adjacent to tumors is largely not well-known. This represents a blind spot, as evidence from many cancer types suggests that grossly normal tissue adjacent to tumors can harbor pre-malignant genetic changes [7,8]. This process has been termed field cancerization and studies have proposed that these early mucosal changes precede and may contribute to tumor development [9,10]. In CRC, this phenomenon has been reported at the epigenetic level, with abnormal DNA methylation patterns detected in histologically normal crypts distant from the tumor [11,12]. Also, somatic copy number variations and single nucleotide variants have been described in histologically normal-appearing colonic tissue from cancer patients [7]. However, a comprehensive characterization of the SM profile in normal colorectal tissue adjacent to tumor, including analysis of which mutations are shared between tumor and normal tissue and what functional consequences they may carry, has not been reported.

As the most commonly mutated gene in CRC, *APC* acts as the gatekeeper of Wnt/β-catenin signaling in the colon [13]. Biallelic inactivation of *APC* is one of the earliest signs of developing CRC and serves as the primary driver of heritable polyposis syndromes like Familial Adenomatous Polyposis [14,15]. *APC* mutations lead to accumulation of β-catenin in the cytoplasm and subsequently in the nucleus that leads to transcriptional activation of Wnt target genes that promote rapid proliferation and cell dedifferentiation [16,17].

CRC development is also associated with disruption of the PI3K/AKT pathway, stress-response signaling, chromatin remodeling, and DNA damage repair systems [2,18]. *APC*, *MAP3K1*, *PIK3CA*, *PTEN*, *ARID1A*, *BRCA2*, and *KMT2C* represent some key factors of these pathways [19,20,21] and are the primary focus of this study.

This study addresses this previously noted blind spot by performing mutational profiling of paired tumor and histologically normal resected colon DNA compared to corresponding WB germline DNA in a cohort of 61 non-metastatic CRC patients, using a custom NGS hybridization capture panel. We characterized the SM profile of normal colorectal tissue, identified mutations shared with tumor tissue, and examined the functional consequences of various mutations in normal tissue on differential gene expression in CRC versus normal tissue using a two-way ANOVA interaction model. Lastly, we explored the implications of our findings for precision medicine, with particular attention to *MTHFD1L* and *MTHFD2* as candidate therapeutic targets in CRC in patients with *APC*-mutated adjacent normal tissue.

## 2. Materials and Methods

### 2.1. Study Design and Patient Cohort

For this study, we included 61 non-metastatic CRC (M = 34, F = 27) patients without chemo- or radiotherapy, who had surgical intervention at Bangabandhu Sheikh Mujib Medical University (BSMMU), Dhaka, Bangladesh, at different times, from December 2009 to May 2016. As part of surgical treatment, the tumor, along with adjacent normal colon tissue, was resected. For the molecular assay, part of the tumor tissue and part of adjacent normal colon tissue (5–10 cm away from tumor) were collected. For each tissue sample, one part was preserved as fresh frozen, and the other part was preserved in RNAlater, an RNA stabilization solution (Invitrogen by Fisher Scientific, Waltham, MA, USA), and kept frozen for gene expression study. At the same time, WB was also collected in EDTA tubes. Samples were shipped on dry ice to the University of Chicago and stored at −80 °C until DNA and RNA extraction.

### 2.2. Tissue Collection and DNA Extraction

Three biological sources were processed for each patient: resected CRC tumor tissue (*n* = 61), adjacent normal colonic tissue (*n* = 61) that was surgically resected along with the tumor, and peripheral whole blood (*n* = 61). DNA was extracted from the fresh frozen tissue using the Puregene Core kit (Qiagen, Germantown, MD, USA). RNA was also extracted from tissue preserved in RNAlater using the RiboPure kit (Qiagen, Germantown, MD, USA) following the manufacturer’s recommended protocol. We used the QIAamp^®^ Blood Mini Kit (Qiagen, Germantown, MD, USA) for whole blood DNA extraction.

### 2.3. Next-Generation Sequencing

For whole genome library preparation, we used the sparQ DNA Frag & Library Prep Kit (Quantabio, Beverly, MA, USA). For tissue and whole blood DNA, we used 35 ng of input DNA. We optimized DNA fragmentation at 32 °C for 14 min. A custom-designed capture panel mainly targeting 32 known cancer-relevant genes, such as CRC driver genes and genes involved in DNA repair, epigenetic regulation, cell-cycle control, and signaling pathways was used for library enrichment. For the enrichment step, we followed the Twist Target Enrichment Standard Hybridization v2 Protocol (Twist BioSciences, South San Francisco, CA, USA). Sequencing was performed on an Illumina NovaSeq-X platform (San Diego, CA, USA) using paired-end 150 bp sequencing.

Raw sequencing reads were aligned to the human reference genome (hg19) using CLC Genomic Workbench 25.0 (https://digitalinsights.qiagen.com/; accessed on 1 July 2026). Somatic variant calling was performed using CLC genomics for each tissue sample against matched whole blood DNA used as the germline to identify somatic mutations. The built-in workflow removed germline variants found in public databases (dbSNP, 1000 genome project, dbSNPs Commons and HapMap). The median sequencing read coverage (R1 and R2) for the mutations was ~2000× (2163× for adjacent normal tissue and 1989× for CRC tissue). Mutation calls were filtered using a minimum coverage of 30×, a minimum variant allele frequency of 1%, a minimum average base quality of 30, and a minimum Phred scale quality score of 120. After filtering out the overlapping R1 and R2 reads, the median coverage of mutation loci was 1430× for the normal tissue and 1382× for the CRC tissue.

### 2.4. Differential Gene Expression Analysis

We utilized the gene expression data for a subset of these samples (*n* = 46) which were available to us from our previous studies [22,23,24,25,26]. Tumor and normal samples from the same individuals were processed on the same microarray chip (Illumina HT12 v4 BeadChip, Illumina Inc, San Diego, CA, USA). For cRNA synthesis, the Illumina^®^ TotalPrep RNA Amplification Kit (Ambion, a part of Life Technologies Corporation, Carlsbad, CA, USA) was used. To assess the functional effect of SM statuses detected in normal colorectal tissue on differential gene expression between CRC and adjacent normal tissue, a two-way ANOVA model was applied. The model included Tissue type (CRC vs. normal) and an interaction term Tissue * mutation status (mutation present [1] vs. absent [0]). The *p*-value of the interaction term identified genes where the expression difference between CRC and normal tissue depended significantly on a certain mutation status.

In Gene Ontology (GO) enrichment analysis, we tested if the genes found to be differentially expressed fell into a GO category more frequently than expected by chance. A chi-square test was used to compare the “number of significant genes from a given category/total number of significant genes” vs. “number of genes on the microarray chip in that category/total number of genes on the chip”. For the enrichment analysis, the probe sets were considered statistically significant if they met two criteria: an FDR-adjusted ANOVA interaction *p*-value < 0.05 and an absolute fold change of >2 in at least one comparison group. Fold change values represented the ratio of expression in CRC relative to normal tissue, with positive and negative values denoting up- and down-regulation, respectively. These statistical analyses were performed using Partek Genomic Software v7.0 (https://www.partek.com/partek-genomics-suite/, accessed on 14 February 2026).

## 3. Results

### 3.1. Patient Cohort and Sample Characteristics

Our patient cohort consisted of 61 patients with non-metastatic CRC: 34 were male (55.7%) and 27 were female (44.3%), with a mean age of 45.5 years (SD 13.7). Most resected tumors were left-sided (*n* = 54, 88.5%) and adenocarcinoma was the predominant histological type (*n* = 52, 85.2%). Patient and tumor characteristics are summarized in Table 1.

### 3.2. Somatic Mutation Profile in Normal Colorectal Tissue Adjacent to Tumor

**Figure 1:**
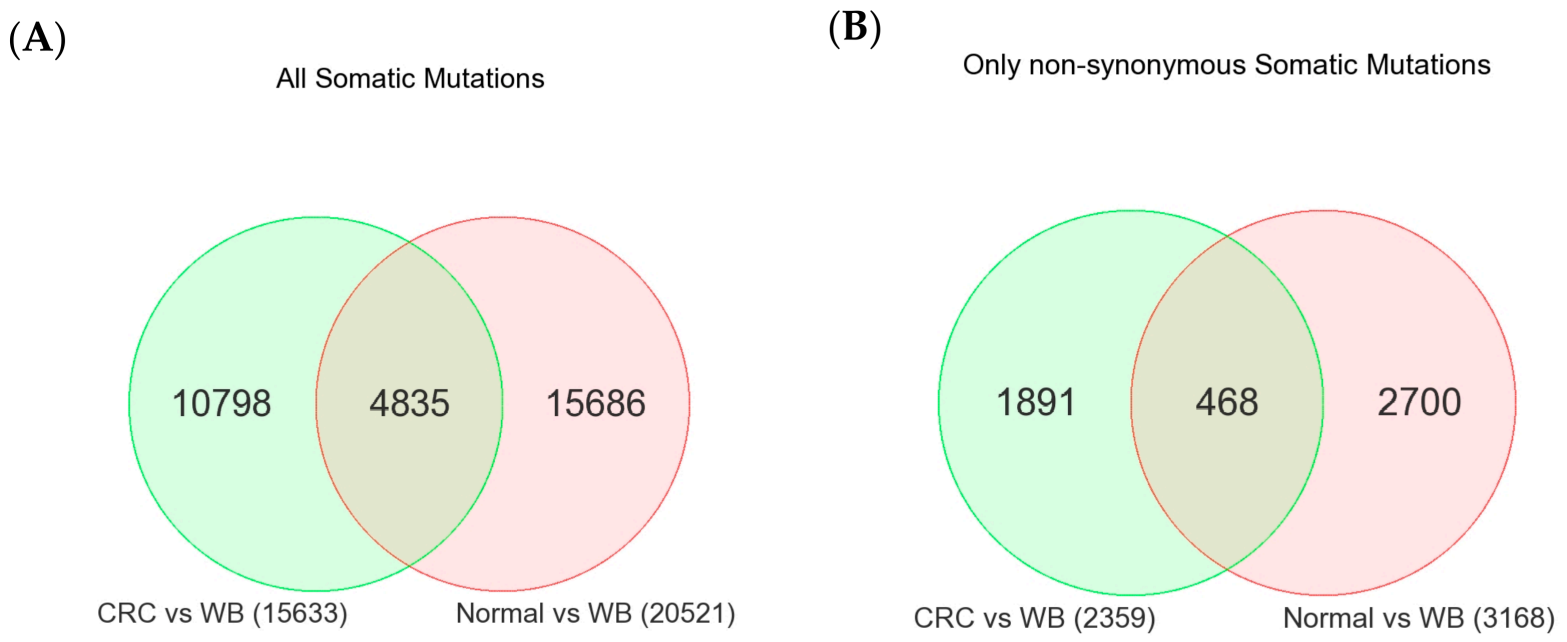
Venn diagrams showing the overlap of somatic mutations identified in the CRC tissue vs. WB and Normal tissue vs. WB comparisons. (**A**) All somatic mutations. A total of 4835 mutation sites were shared, with 10,798 mutations unique to CRC tissue and 15,686 unique to adjacent normal tissue. (**B**) Non-synonymous somatic mutations only. A total of 468 mutation sites were shared (potentially malignant non-synonymous SMs), with 1891 mutations unique to CRC tissue and 2700 unique to normal tissue.

SMs were identified in both the tumor and adjacent normal tissue of all 61 patients. Notably, the total number of SMs detected in normal colonic tissue was almost similar to that observed in tumor tissue. More than 23% of those identified in normal tissue were also detected in the tumor tissue (see Figure 1A). These shared mutations (also termed “potentially malignant” mutations throughout this paper) were identified in 61 of 61 patients (100%) when considering all mutation types, indicating that all CRC patients may harbor at least some SMs in their normal colon that are also present in their tumor. Restricting to non-synonymous mutations only (see Figure 1B), 57 of 61 patients (93.4%) had at least one potentially malignant non-synonymous SM in their adjacent normal tissue.

**Figure 2:**
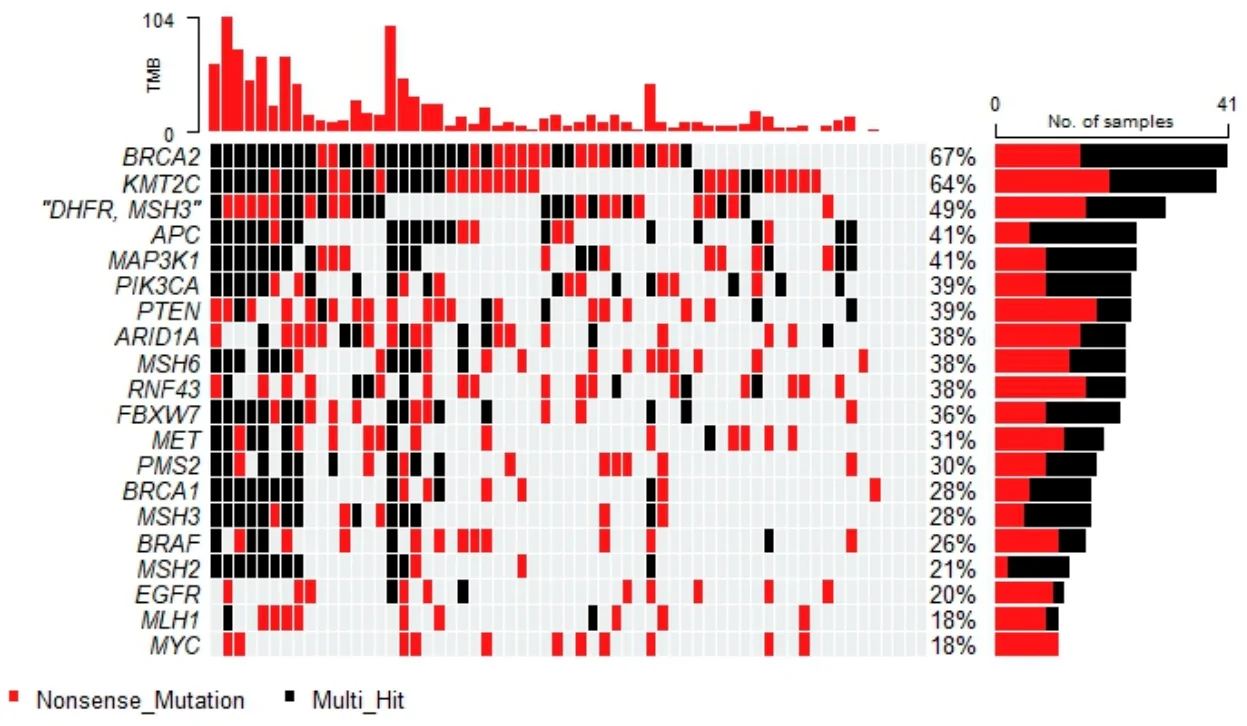
Oncoplot showing the top 20 genes which show non-synonymous potentially malignant somatic mutations in normal tissue adjacent to tumor tissue in all patients in the cohort (*n* = 61). These mutations were also found in CRC tissues shown in the Venn diagram in Figure 1B. Columns represent individual patients and rows represent genes. Red squares represent single nonsense mutations and black squares indicate multiple hits within the same gene. The top bar plot shows mutational burden per patient. The right bar plot shows frequency of mutations across patients.

Considering only the potentially malignant non-synonymous SMs in normal tissue that are also found in CRC tissue (see the overlapping 468 unique loci in Figure 1B), the most frequently affected genes across the cohort are summarized in an oncoplot shown in Figure 2. *APC* was mutated in the normal tissue of 41% of patients, ranking fourth overall after *BRCA2*, *KMT2C*, and *DHFR*. Mismatch repair gene *MSH6* and chromatin remodeling gene *ARID1A* were also commonly involved, as were key oncogenic signaling pathway genes *PIK3CA*, *PTEN*, and *MAP3K1*. In this study, we have intentionally focused only on the SMs in normal colon tissues and plan to discuss the SMs in the CRC tissue in a separate study.

### 3.3. Using Matched Whole Blood Germline DNA as a Reference for SNV Calling

**Figure 3:**
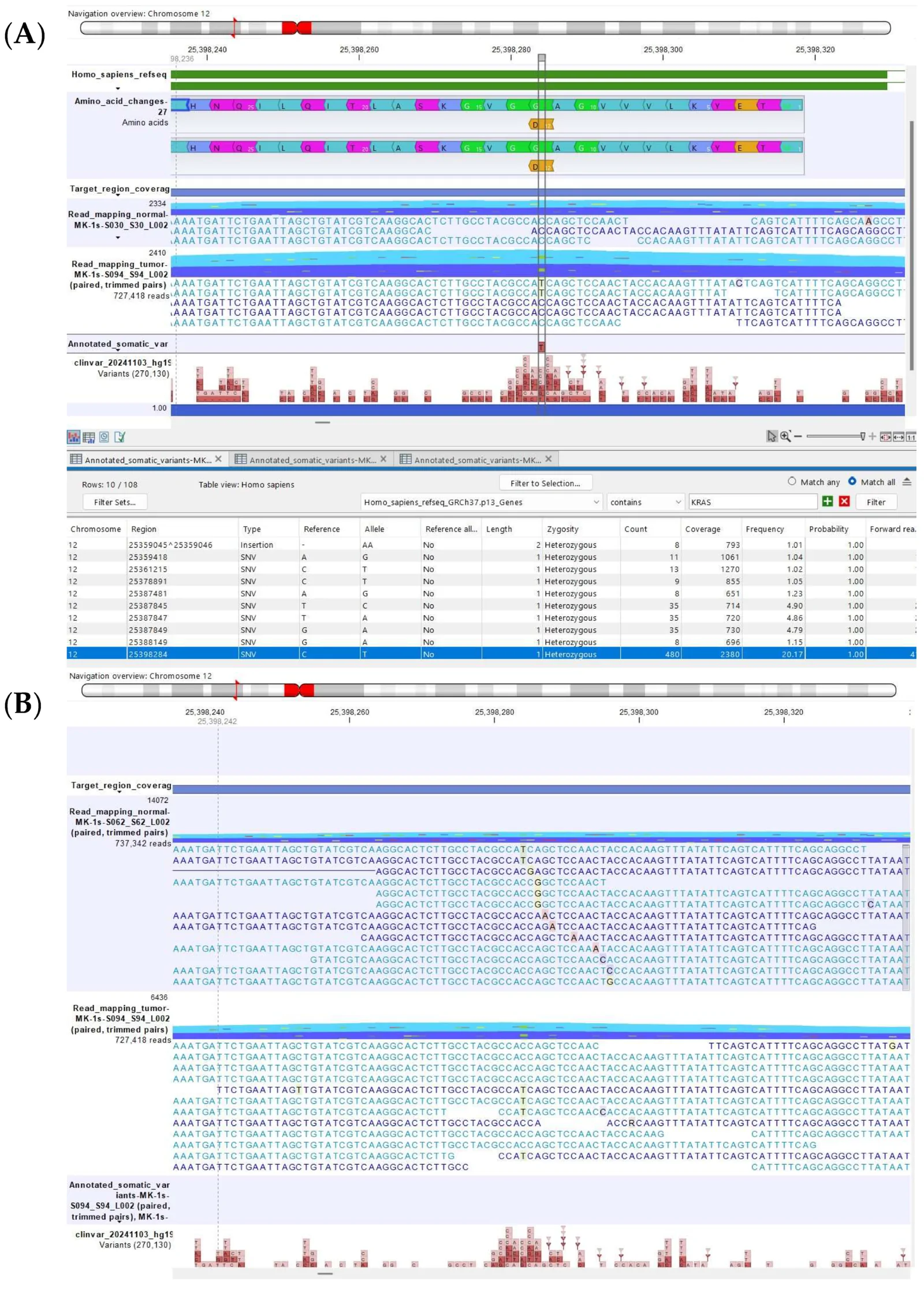
Whole blood as a germline reference prevents false negatives in variant calling. (**A**) *KRAS* p.Gly12Asp mutation was detected at 20.17% allele frequency in tumor tissue of a patient C102 when compared against matched whole blood, confirmed as a somatic mutation. (**B**) The same site in patient C102 when compared against normal colon tissue. The mutation was absent from calls because it was present in both tumor and normal tissue and was filtered as a non-somatic event, presenting a false negative.

This study presented the important role of using matched WB germline DNA as a reference during somatic variant calling in CRC, illustrated in Figure 3. A patient had a *KRAS* p.Gly12Asp substitution mutation detected at a 20.17% allele frequency, seen in Figure 3A, in tumor tissue when compared against matched whole blood. In Figure 3B, this appeared absent from somatic variant calls when adjacent normal colon tissue was used as the reference instead. This is because the mutation was present in both tumor and normal colon and was filtered as a non-somatic event, causing a false negative.

### 3.4. Cancer-Driver Mutations in Normal Colorectal Tissue Adjacent to Tumor

We performed cross-referencing of the SMs detected in normal tissue against the IntOGen database [27] of driver genes in colon adenocarcinoma (COAD) and rectum adenocarcinoma (READ), which documents driver mutations identified in cancer genomics cohorts including The Cancer Genome Atlas (TCGA) and the International Cancer Genome Consortium (ICGC).

IntOGen reports a total of 849 driver mutations located at 723 unique genomic coordinates in CRC (COAD + COREAD) from 15 genes. We sequenced 10 of those 15 genes in this study. In our 61 patients, we found that 31 of them (50.8%) had at least one of these known driver mutations in their normal colon tissue. Also, driver mutations in 8 of the 10 genes sequenced in this study were detected in normal colon tissue. It may be noted that if tumor tissue was compared to adjacent normal tissue for SM detection, these known driver SMs would have been missed in 50% of the patients. The eight genes with known driver mutations in normal colon tissue are shown in Table 2. Among these eight genes, driver mutations in *APC* were the most frequently encountered.

**Figure 4:**
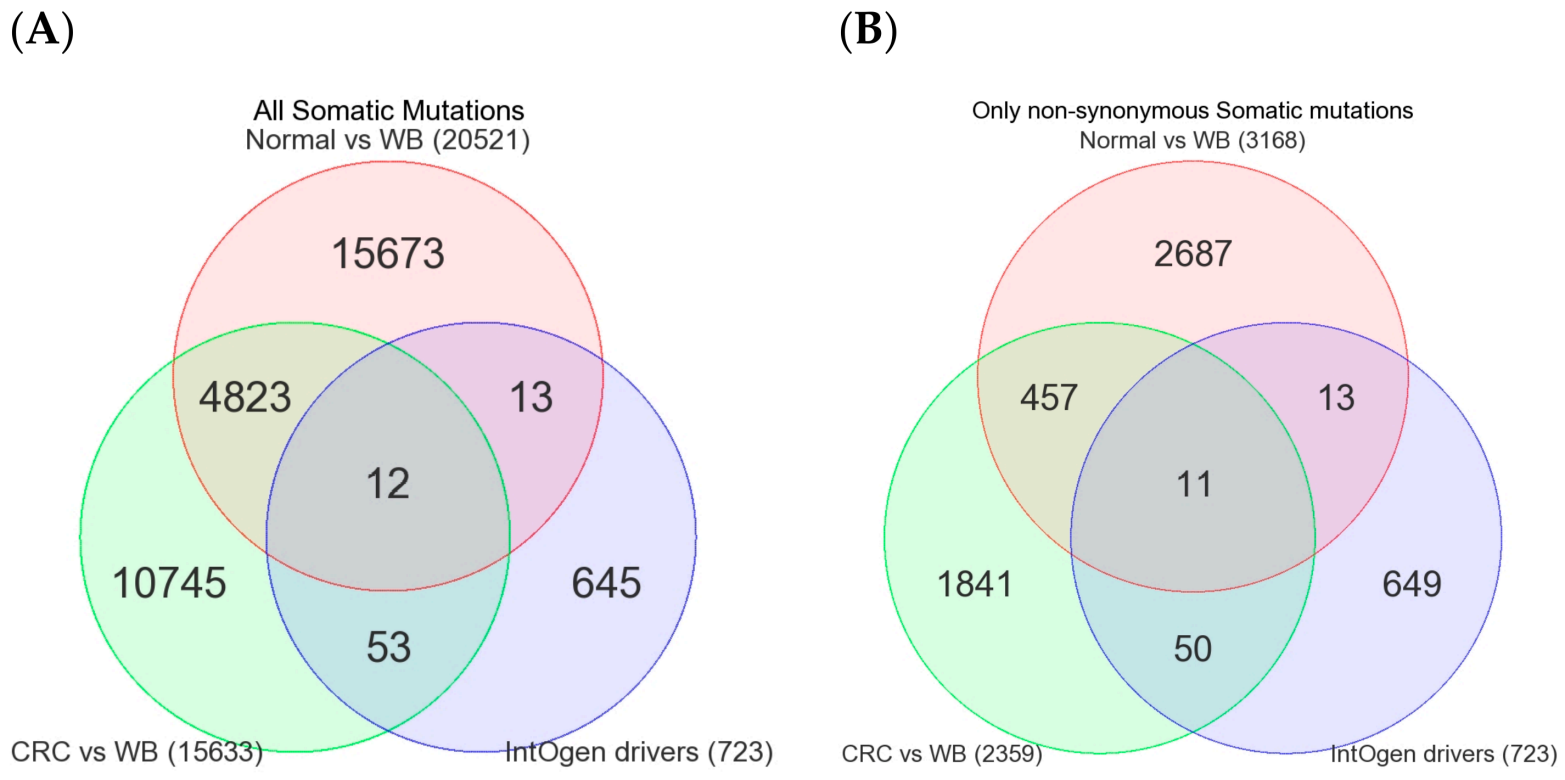
Overlap of somatic mutations with IntOGen CRC driver mutation positions. (**A**) All somatic mutation sites identified in Normal tissue vs. WB and CRC tissue vs. WB compared to 723 IntOGen driver gene coordinates. (**B**) Same analysis as Figure 4A, but considering only non-synonymous mutations.

To further characterize the distribution of driver mutations across our cohort, we compared identified somatic mutation sites from both tissue comparisons against the 723 IntOGen driver coordinates, seen in Figure 4. Across all somatic mutations, 65 driver mutation positions were detected in the CRC vs. WB comparison and 25 in the Normal vs. WB comparison (Figure 4A). Notably, 12 driver mutation sites were shared between CRC and adjacent normal tissue, which may have been missed if WB was not used as a reference. Considering only the non-synonymous variants, we retained 61 and 24 driver mutation positions, respectively (Figure 4B).

### 3.5. Functional Consequences of Potentially Malignant Non-Synonymous Somatic Mutations in Normal Tissue (That Were Also Found in CRC Tissue)

In the next steps, we examined the functional significance of these potentially malignant non-synonymous SMs in normal colorectal tissue on the differential gene expression in tumors. By differential expression, we mean the gene expression comparisons between CRC tumor tissue and adjacent normal colon tissue, stratified by whether the patient had the specified non-synonymous SM in the normal tissue adjacent to the CRC or not. We examined the most frequently mutated genes shown in the oncoplot above (Figure 2). All subsequent gene expression analysis in Section Impact of Potentially Malignant Non-Synonymous *APC* Mutation Status in Normal Colon on Differential Gene Expression and Section 3.6, Section 3.7, Section 3.8, Section 3.9, Section 3.10 and Section 3.11 was performed only on 46 samples with available gene expression data. The mutational statuses of these 46 samples for the most frequently mutated genes are shown in Supplementary Table S1. The details of ANOVA results for all analyzed genes (*APC*, *MAP3K1*, *PIK3CA*, *PTEN*, *ARID1A*, *BRCA2* and *KMT2C*) are presented in Supplementary Tables S2–S8. Throughout all the tables, an FDR-adjusted interaction *p*-value < 0.05 and an absolute fold change of >2 in at least one comparison group was used to identify genes for which the difference in expression between CRC and normal tissue depended significantly on mutation status.

#### Impact of Potentially Malignant Non-Synonymous *APC* Mutation Status in Normal Colon on Differential Gene Expression

**Figure 5:**
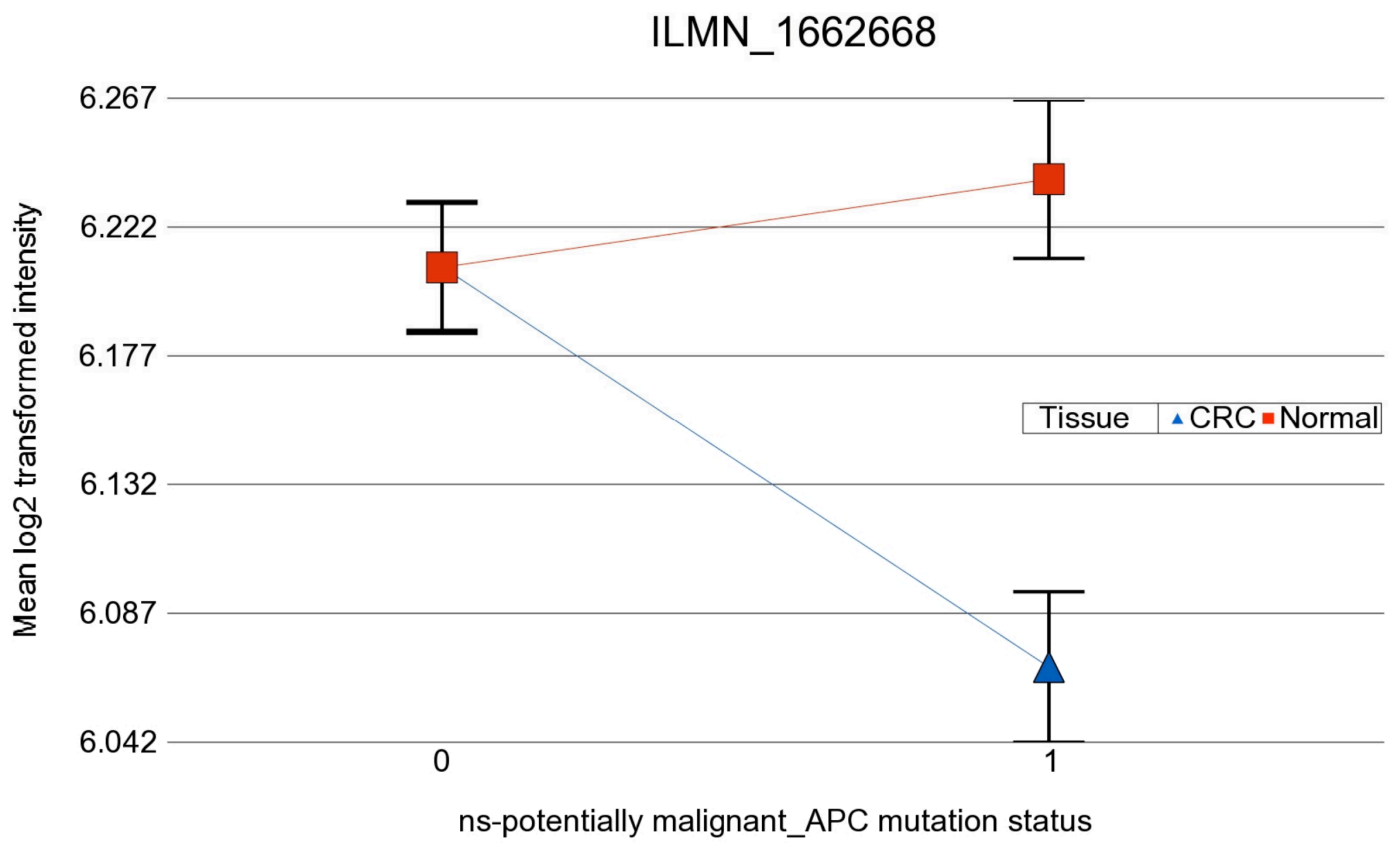
Differential expression of *APC* stratified by patients who had potentially malignant *APC* non-synonymous SMs in normal colon tissue (shown as 1 in the x-axis), and those who did not have such mutation in their normal colon tissue (shown as 0 in the x-axis). Only the patients with such *APC* somatic mutations in normal colon tissue showed significantly lower *APC* expression in CRC tumor tissue (blue), while adjacent normal colon tissue expression (red) remained similar between groups.

Interaction analysis for *APC* showed that patients with a non-synonymous potentially malignant SM in *APC* in their normal colon tissue had significantly lower *APC* expression in their CRC tumor tissue. Given that *APC* is a well-established tumor suppressor, this finding suggested that it may be associated with early functional alterations in normal tissue. This interaction analysis is illustrated in Figure 5.

To assess whether the presence of *APC* mutation in adjacent normal colon tissue changes the transcriptomic response of CRC versus normal tissue, a two-way ANOVA interaction model (Tissue * *APC* mutation status) was applied. This identified several genes with statistically significant interactions, indicating that the magnitude of differential expression in CRC versus normal tissue is significantly different between patients with and without *APC* mutation in their adjacent normal colon. Fold changes (FCs), confidence intervals (CIs), and interaction *p*-values for the findings in this section are summarized in Table 3. The complete statistical analysis of all the genes is shown in Supplementary Table S2. This clearly showed that the magnitudes of differential gene expression (CRC vs. normal colon tissue) were significantly modified in the presence or absence of the SM in *APC* in the normal tissue.

Down-regulation of several genes was dependent on the presence of *APC* mutations. For example, *SLC26A3* was down-regulated in either case, but the magnitude of down-regulation was significantly more marked in patients whose adjacent normal colon tissue had non-synonymous potentially malignant *APC* mutation (see Table 3). Similar effects were seen in *SELENBP1*, *KRT20*, and *CXCL14*.

Up-regulation of some genes was dependent on the presence of the *APC* mutation. For example, *MMP1* and *MMP12* were up-regulated in either case, but the magnitude of up-regulation was significantly more marked in patients whose adjacent normal colon tissue had non-synonymous *APC* mutation. *MMP7* was up-regulated in either case but, uniquely, with a stronger effect in patients with wildtype *APC* in normal tissue.

**Figure 6:**
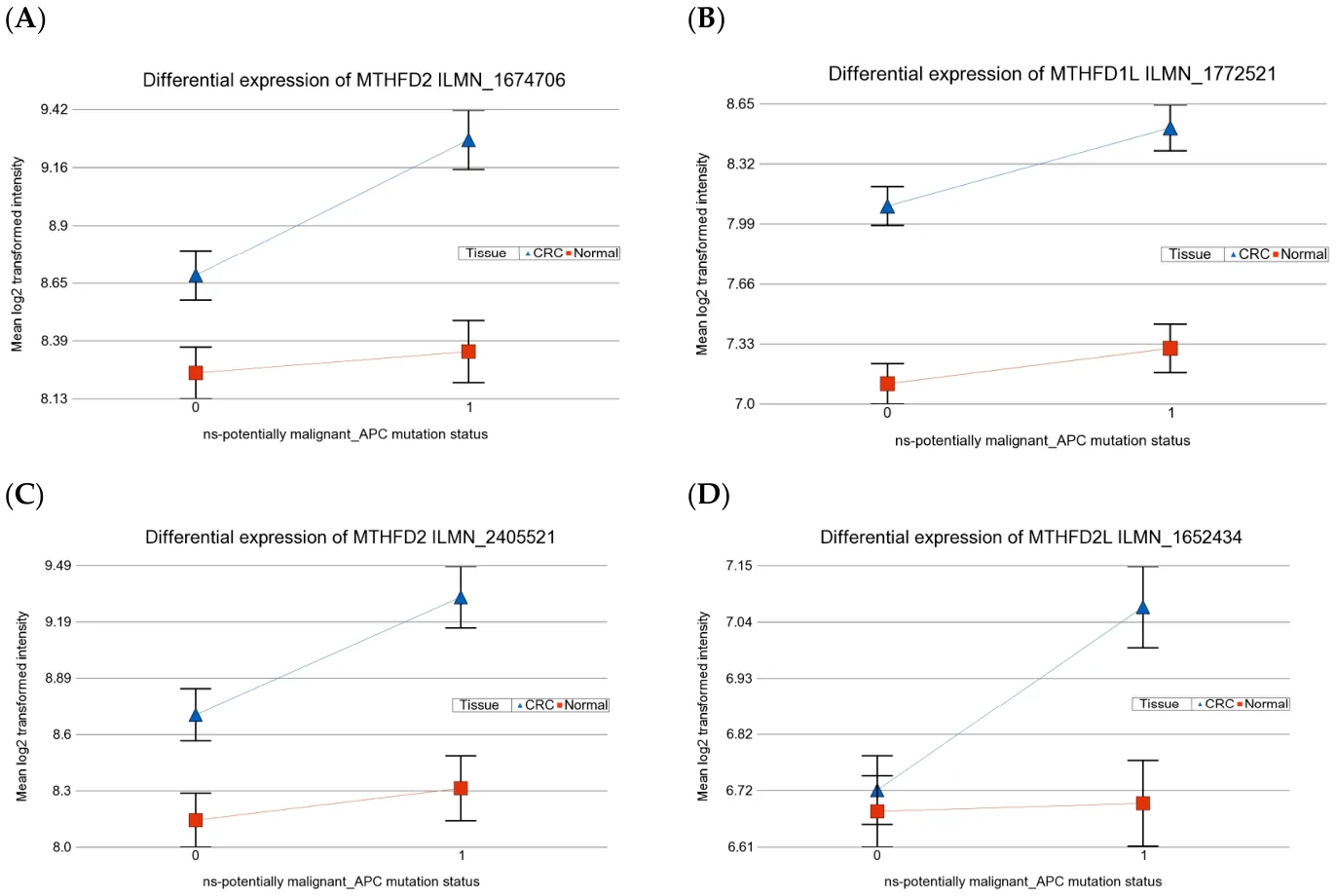
Differential expression of (**A**) *MTHFD2*, (**B**) *MTHFD1L*, (**C**) an alternative probe for an *MTHFD2* isomer, and (**D**) *MTHFD2L* grouped by *APC* non-synonymous somatic mutation status in normal colon tissue, with 0 indicating no mutation and 1 indicating the presence of an *APC* mutation. Patients with *APC* somatic mutations in adjacent colon tissue regularly showed increased expression of *MTHFD* genes in CRC tumor tissue (blue) relative to normal colon tissue (red).

Among the genes significantly up-regulated in CRC versus normal tissue with a significant interaction for *APC* mutation status, *MTHFD1L* and *MTHFD2* were noted as possible candidates for targets in precision medicine. In our data, both were up-regulated in CRC tissue relative to normal tissue in tumors with both *APC* wild and *APC* mutant adjacent tissue, with a significantly marked increase in expression in the group with *APC*-mutated adjacent normal colon. The differential expression for folate-cycle-related *MTHFD* genes is illustrated in Figure 6A–D. Notably, *MTHFD2* exhibited the largest fold change in expression. Detailed two-way ANOVA analyses for the *MTHFD* genes are presented in Supplementary Table S9.

**Figure 7:**
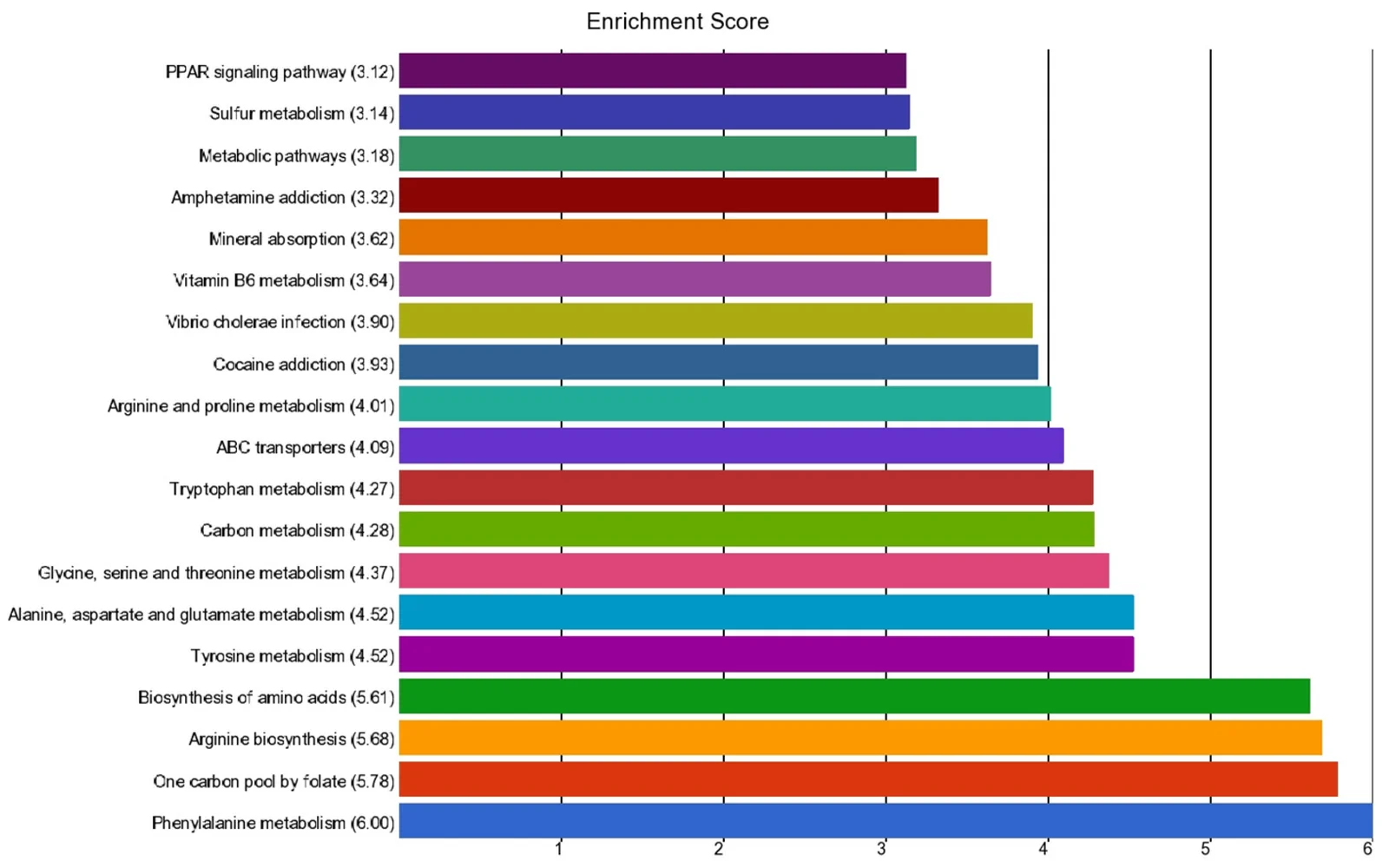
Pathway enrichment analysis on genes with significant differential expression in tumors caused by *APC* mutation in adjacent normal tissue. Bars represent enrichment scores, with corresponding scores shown in parentheses.

Pathway enrichment analysis was performed on genes with significant differential expression in tumors caused by *APC* mutation in adjacent normal tissue, as seen in Figure 7. Enriched pathways were predominantly associated with amino acid metabolism, one-carbon metabolism, and transport processes.

### 3.6. Functional Consequences of Potentially Malignant Non-Synonymous MAP3K1 Somatic Mutation in Normal Tissue

We identified 57 probe sets with statistically significant interactions. The statistical analysis for this section is summarized in Supplementary Table S3. The most affected gene was *CA1*. In tumors with *MAP3K1*-wildtype adjacent normal tissue, *CA1* was already down-regulated in CRC (FC = −17.7, 95% CI −41.1 to −7.6). In patients with *MAP3K1* mutation in normal tissue, this down-regulation was increased (FC = −88.6, 95% CI −213.0 to −36.8; interaction *p* = 0.011). Similarly, *JCLN*, *CA4*, and *SLC26A3* showed significantly greater down-regulation in tumors with *MAP3K1*-mutated adjacent normal tissue.

Consistent with this, the tight junction gene *CLDN8* and the mucin-related gene *MUPCDH* also showed greater down-regulation in tumors with *MAP3K1*-mutated adjacent normal tissue, suggesting diminished epithelial barrier integrity. In contrast, some genes were more strongly up-regulated in tumors with *MAP3K1*-mutated adjacent normal tissue, including *COL11A1* and *ESM1*. Interestingly, a direction reversal was seen in *LYZ* (wildtype vs. mutant FC = −1.0 vs. +2.0; interaction *p* = 0.004). Abbreviations for major gene names and terms used throughout this paper are defined in Supplementary Table S10.

### 3.7. Functional Consequences of Potentially Malignant Non-Synonymous PIK3CA Somatic Mutation in Normal Tissue

The ANOVA identified 57 probe sets with statistically significant interactions. The statistical analysis for this section is summarized in Supplementary Table S4. Several extracellular matrix remodeling genes were strongly up-regulated in CRC with *PIK3CA*-mutated adjacent normal tissue. For example, *MMP1* showed increased up-regulation in tumors with *PIK3CA*-mutated adjacent normal tissue and *MMP3* (FC = +9.5 vs. +26.0; interaction *p* = 0.016) showed a greater increase. *COL8A1*, *THBS2*, and *CTHRC1* were also significantly increased. Another prominent group of similarly affected genes involved inflammatory cytokines. This included IL8 (FC = +18.4 vs. +30.9; interaction *p* = 0.019), IL6, and IL24.

### 3.8. Functional Consequences of Potentially Malignant Non-Synonymous PTEN Somatic Mutation in Normal Tissue

We found 37 probe sets with statistically significant interactions. The statistical analysis for this section is summarized in Supplementary Table S5. The main pattern in tumors with *PTEN*-mutated adjacent normal tissue was increased up-regulation of oncogenic and pro-invasive genes. *MMP11* and *SALL4* showed the greatest effect. An exception was *REG1A*, which showed the highest fold change of any gene in the *PTEN* analysis, showing a reduced up-regulation in tumors (FC = +43.8 vs. +14.1; interaction *p* = 0.007). In contrast, *PTEN* mutations in adjacent normal tissue were associated with increased down-regulation of *ABCA8*, *PPARGC1A*, and *LRRN2* in tumors.

### 3.9. Functional Consequences of Potentially Malignant Non-Synonymous ARID1A Somatic Mutation in Normal Tissue

The analysis identified 12 probe sets with statistically significant interactions. The statistical analysis for this section is summarized in Supplementary Table S6. *ARID1A* mutation interactions were mainly characterized by lessening of gene suppression. *CKB*, *MUPCDH*, *MAOA*, *DENND2A*, *CA12*, and *TP53INP2* all showed similarly reduced down-regulation in tumors with *ARID1A*-mutated adjacent normal tissue. Exceptions included *NAAA*, which showed increased down-regulation (FC = −1.8 vs. −2.2; interaction *p* = 0.045), and *TTYH3*, which showed increased up-regulation (FC = +1.4 vs. +2.3; interaction *p* = 0.033).

### 3.10. Functional Consequences of Potentially Malignant Non-Synonymous BRCA2 Somatic Mutation in Normal Tissue

We identified nine probe sets with statistically significant interactions. The statistical analysis for this section is summarized in Supplementary Table S7. The statistically most significant interaction was *CLEC3B* (FC = −3.2 vs. −2.3; interaction *p* = 0.033), exhibiting reduced down-regulation in tumors with *BRCA2*-mutated adjacent normal tissue. *SLC25A34*, *CLIC5*, and *MUPCDH* all showed similar patterns. Interestingly, metallothionein genes *MT1A*, *MT1X*, and *MT1E* instead showed increased down-regulation patterns (MT1A: FC = −1.8 vs. −2.4, *p* = 0.032; MT1X: FC = −1.5 vs. −2.2, *p* = 0.036; MT1E: FC = −2.4 vs. −3.0, *p* = 0.047).

### 3.11. Functional Consequences of Potentially Malignant Non-Synonymous KMT2C Somatic Mutation in Normal Tissue

The ANOVA identified 34 probe sets with statistically significant interactions. The statistical analysis for this section is summarized in Supplementary Table S8. Among the genes with increased down-regulation in tumors with *KMT2C*-mutated adjacent normal tissue, *FLJ21511* and *TSPAN7* showed similar effects. *UGT2A3* and *UGT1A10* showed increased down-regulation to a lesser extent. Among the genes with increased up-regulation in the same group, *CDH3*, *SLC7A5*, and *ETV4* showed the greatest effects. An interesting finding was *CCL8*, which had a direction reversal, showing slight up-regulation in tumors with *KMT2C*-wildtype adjacent normal tissue (FC = +1.4) and significant down-regulation in tumors with *KMT2C*-mutated normal tissue (FC = −2.4; interaction *p* = 0.026).

### 3.12. Cross-Gene Trends and Recurrent Genes

Altogether, the two-way ANOVA interaction analyses across all seven observed mutated genes provided several patterns. Extracellular matrix remodeling genes recurred often across multiple mutations. *MMP1* up-regulation was increased in tumors with both *APC*- and *PIK3CA*-mutated adjacent normal tissues, while *MMP3* was the most strongly increased in tumors with *PIK3CA*-mutated adjacent normal tissue. *MMP11* showed the greatest up-regulation in the *PTEN* analysis and *COL11A1* was increased in *MAP3K1* analysis. *SERPINE1* appeared in two interaction lists, exhibiting increased up-regulation in tumors with *PIK3CA*-mutated normal tissue and decreased up-regulation in *KMT2C*-mutated normal tissue.

Several metabolic genes recurred across the cohort. *PPARGC1A* showed increased down-regulation in tumors with both *MAP3K1*- and *PTEN*-mutated adjacent normal tissue. *PFKFB4* showed up-regulation in *MAP3K1* analysis. *GPT2* was up-regulated in tumors with both *PTEN*- and *KMT2C*-mutated normal tissue. *SLC7A5* showed up-regulation in *KMT2C* analysis.

Other noted recurrently interacting genes included *SLC26A3* which showed increased down-regulation in both *APC* and *MAP3K1* analyses. *TSPAN7* appeared in both the *PTEN* and *KMT2C* analyses with increased down-regulation. *MUPCDH* appeared with decreased down-regulation in both *ARID1A* and *BRCA2* analyses.

## 4. Discussion

This study provides one of the first comprehensive characterizations of the SM profile of apparently normal colorectal tissue adjacent to tumors in CRC patients, using matched WB germline DNA as the reference. It was demonstrated that apparently normal looking colon in CRC patients contains a significant burden of potentially malignant non-synonymous SM, with total mutation counts comparable to those seen in adjacent tumor tissue when both are compared to matched germline DNA. This suggests that apparently normal tissue adjacent to CRC is not always genomically unaltered and may contain potentially malignant SMs.

Field effect (also known as field cancerization or field carcinogenesis) refers to the presence of genetically altered, preneoplastic cells within a tissue area that appears histologically normal, which predisposes the tissue to the development of multiple independent tumors or local recurrence. The biology of field cancerization in CRC has been primarily studied with epigenetics, such as methylation of genes in histologically normal CRC crypts [11,12]. Our data aimed to expand this concept to the genomic level by including driver-gene-level SM. It may be noted that prior studies of colorectal precursor lesions and circulating cell-free DNA emphasize the early accumulation of driver mutations such as *APC*, *KRAS*, and *TP53* [28,29].

At least one potentially malignant non-synonymous mutation was identified in 57 of 61 patients (93.4%). This high prevalence suggested that clonal expansions with these mutations may precede the tumor formation in most CRC patients and can serve as signs of cancer risk in the colon before detectability with histopathology. This has an important practical implication which is that a direct comparison between tumor and normal tissue, without a germline reference, would fail to detect the potentially malignant shared mutations. As demonstrated by our findings in Figure 4, because these mutations are present in both tissue sections, a standard tumor-versus-normal paired comparison would fail to detect these SMs in the tumor tissue.

The clinical significance of detecting these SMs in normal colon tissue was further supported by evaluating them against established CRC driver genes. Cross-referencing our findings against the IntOGen database of driver mutations showed that eight established COAD/READ driver genes were recurrently mutated in the normal colon tissue adjacent to tumors in our cohort. This supported our designation of these normal tissue mutations as potentially malignant.

After variant calling, 31,980 mutations in total were identified across 61 patients, of which 24,472 (76.5%) were single nucleotide variants (SNVs). IntOGen documented positions for 723 driver mutation positions across 15 CRC driver genes. Filtering our SNV calls for those which overlapped with these positions retained 47 variants across 31 patients, representing 26 unique variants across eight driver genes, seen in Table 2.

The presence of driver mutations in the adjacent normal colon tissue was associated with significantly modified gene expression patterns in the matched CRC tumor tissue. Being matched samples, the mutations detected in normal colon tissue were also identified in the matched tumor tissue, confirming that these shared mutations are present in both groups. This finding is consistent with the concept of field cancerization in that such mutations arising in the normal epithelium may represent the earliest molecular events in CRC development, before histological transformations [9,10]. Reconfigurations of genotype-methylation dynamics may contribute during initial field events in the normal colon [30]. In our data, the significant increase in *SLC26A3*, *SELENBP1*, *KRT20*, and *CXCL14* down-regulation, along with greater *MMP1* and *MMP12* up-regulation in tumors with *APC*-mutated adjacent normal tissue, collectively suggested a more dedifferentiated and invasive tumor microenvironment present. We acknowledge that alternative interpretations exist, such as the SM in adjacent normal tissue reflecting a broader colonic genomic instability, and that the precise causal mechanism warrants further investigation.

*SLC26A3* encodes a chloride/bicarbonate transporter that is expressed in normal colon and helps maintain epithelial barrier integrity [31]. In our cohort, its down-regulation in CRC and further suppression in tumors with *APC*-mutated adjacent normal tissue is consistent with the degradation of the normal colonic epithelium. Similarly, *SELENBP1* is a tumor suppressor in CRC whose expression is diminished further down the adenoma-carcinoma sequence [32]. Its increased down-regulation in tumors with *APC*-mutated adjacent normal tissue may suggest an effect on cell signaling that accelerates the epigenetic silencing of *SELENBP1* during advanced colon cancer [33]. Also, *KRT20* is a marker of fully developed colon cells [34] and its increased down-regulation in tumors with *APC*-mutated adjacent normal tissue suggests that dedifferentiation occurred. This may be due to increased β-catenin signaling, which promotes tumor cells to revert to a stem-like growth state [35]. Additionally, *CXCL14* is a chemokine with tumor-suppressive properties in CRC with anti-angiogenic and immune-modulatory properties [36]. Its increased down-regulation in tumors with *APC*-mutated adjacent normal tissue may suggest a defective anti-tumor response, allowing for immune evasion.

Up-regulation of some genes was dependent on the presence of the *APC* mutation. For example, *MMP1* is an interstitial collagenase and a driver of tumor metastasis [37]. Its increased up-regulation in tumors with *APC*-mutated adjacent normal tissue may suggest an induction of a more invasive tumor microenvironment, possibly caused by Wnt/β-catenin pathway activation [38]. Similarly, *MMP12* plays a role in extracellular matrix remodeling and tumor angiogenesis [39]. Its increased expression in tumors with *APC*-mutated adjacent normal tissue may be due to a more invasion-permissive microenvironment. *MMP7* is a protease that degrades the basement membrane and allows cells to migrate [40]. Its higher expression in tumors with *APC* wildtype adjacent normal tissue may be due to altered growth signals in the tumor tissue.

Beyond *APC*, the interaction analyses for the other six analyzed genes extend upon this concept. The *MAP3K1* interactions were characterized by their extreme magnitudes, particularly the intense suppression of *CA1* (FC = −88.6) alongside key epithelial transporters *JCLN*, *CA4*, and *SLC26A3* in patients with *MAP3K1*-mutated normal tissue. *CA1* encodes a colonocyte-expressed enzyme that maintains bicarbonate buffering, and its extreme loss here may reflect failure of colonocyte terminal differentiation [41]. The *PIK3CA* interactions were centered on ECM remodeling and cytokine genes, consistent with the biology of PI3K/AKT-driven invasion and inflammatory microenvironment reprogramming [42]. The *PTEN* interactions were notable due to up-regulations of *MMP11* and *SALL4* in patients with *PTEN*-mutated normal tissue. Together, they may be associated with ECM remodeling and aggressive tumor behavior by PI3K pathway hyperactivation, as previously documented for *SALL4* [43]. The *ARID1A* interactions were notable in that the majority of significant interactions were characterized by lessening the suppression of genes, most playing roles in metabolism.

*BRCA2* (67%) and *KMT2C* (64%) were the most frequently mutated genes across our cohort, even exceeding *APC* (41%). The *BRCA2* interaction signature was the only one in this study in which no gene showed net up-regulation in either group. All nine significantly interacting genes were down-regulated in tumors with both *BRCA2*-wildtype and mutated adjacent normal tissue. Most genes showed reduced suppression in tumors with *BRCA2*-mutated adjacent normal tissue, which suggested that *BRCA2* loss in the pre-malignant tissue may counteract gene suppression. Conversely, the three metallothionein genes *MT1A*, *MT1X*, and *MT1E* showed increased suppression in tumors with *BRCA2*-mutated adjacent normal tissue. Because they serve as defense networks against cellular damage [44], this division may reflect how *BRCA2* mutation in normal colon causes a broader epigenetic de-repression effect at most mutation sites, and a specific further silencing of these oxidative stress response genes. In the case of *KMT2C*, the interactions were the most complex of all mutations studied, encompassing increased up-regulation, increased down-regulation, and our observation of a unique *CCL8* direction reversal. This may reflect *KMT2C*-dependent regulation of immune chemokine expression in the tumor [45].

The recurrent ECM remodeling signatures observed across *APC*, *MAP3K1*, *PIK3CA*, and *PTEN* interaction analyses suggest that SM in the adjacent normal colon influences the developing tumor toward a more invasive state.

Our findings around *MTHFD1L* and *MTHFD2* in the *APC* mutant cohort were particularly important in terms of precision medicine. Both enzymes participate in the folate cycle: *MTHFD2* catalyzes conversions of tetrahydrofolate derivatives which carry one-carbon units within the mitochondria, while *MTHFD1L* catalyzes the final step of the pathway, allowing export of one-carbon units into the cytoplasm for nucleotide biosynthesis [46]. Proliferating cancer cells are highly dependent on one-carbon metabolism for DNA replication and cell growth, and it has been documented that cancer cells frequently reactivate and depend on *MTHFD2* to further drive this process [47,48]. Our data further suggests that *MTHFD1L* is up-regulated alongside *MTHFD2* in tumors with *APC*-mutated adjacent normal tissue, pointing to up-regulation of the folate pathway as a characteristic of this group which may serve as a therapeutic target.

Pseudolaric acid A (PAA) is a diterpenoid derived from the root bark of *Pseudolarix amabilis*, a plant used in traditional Chinese medicine. A recent target-identification study using activity-based protein profiling and drug affinity responsive target stability techniques in HeLa cells identified *MTHFD1L* as a direct molecular target of PAA [49]. Given that our data shows significant up-regulation of *MTHFD1L* specifically in tumors with *APC*-mutated adjacent normal tissue, PAA may represent a possible therapeutic agent for this group and its investigation in CRC model systems can be beneficial.

Similarly, the up-regulation of *MTHFD2* in our cohort motivates investigation of direct *MTHFD2* inhibitors. Several small-molecule inhibitors of *MTHFD* genes have shown preclinical success, supporting the therapeutic potential of targeting one-carbon metabolism. Notably, TH9619 is a *MTHFD1*/*MTHFD2* inhibitor that is currently in phase 1/2 clinical trials for advanced solid tumors (NCT07151040) [50]. Preclinical studies indicate that TH9619 kills cancer cells by trapping folate and depleting thymidine, causing DNA damage and replication stress specifically in tumor cells while sparing normal cells [51]. More broadly, methotrexate (MTX) is an established antifolate chemotherapy drug that indirectly inhibits the folate cycle upstream of *MTHFD* genes. Studies show it can act synergistically with other therapies by blocking the folate pathway in cells with high *MTHFR* expression [52,53]. More selectively, DS18561882 is an isozyme-selective compound in preclinical development that inhibits *MTHFD2*, demonstrating in vivo anti-tumor activity [54].

This study had several limitations that should be considered when interpreting the findings. All normal colonic tissue was obtained from CRC patients rather than healthy controls, so we cannot comment on the significance of SMs that may be detected in normal colon tissue of healthy individuals. Our custom hybridization capture panel covered only a defined set of genes and so mutations in other genes were not assessed in normal tissue. Also, our gene expression analyses were limited to only 46 samples with available data. A larger cohort and validation with independent RT-qPCR experiments would further strengthen our observed differential gene expression patterns.

Despite these limitations, this study supports the use of WB germline DNA as a reference during somatic variant calling in CRC and can inform therapeutic strategies in precision medicine. The results provide some molecular genomic basis to consider SM testing from colonoscopic biopsy material in selected high-risk or suspected cases. The use of ideally preserved fresh frozen paired samples (tumor–normal) from the same individuals is a strength of the present study. Because the most frequently mutated genes in normal tissue in our cohort were associated with interaction signatures, these insights motivate further research into how potentially malignant mutations can be targeted.

## 5. Conclusions

This study documents three distinct findings regarding SMs in normal colorectal tissue adjacent to tumors in CRC. First, non-synonymous mutations are present in the normal colon in a vast majority of CRC patients, including driver mutations in established CRC genes. Second, these mutations may be missed if tumor tissue is compared against adjacent normal tissue without the use of a whole blood germline reference. Third, these mutations in normal tissue have significant functional consequences on differential gene expression in CRC and may have relevance to precision medicine. Notably, in patients with *APC*-mutated adjacent normal tissue, differential expressions of *MTHFD1L* and *MTHFD2* may suggest the folate pathway as a potential candidate for targeting.

## Figures and Tables

**Table 1:** Patient and tumor characteristics of 61 CRC patients in the study’s cohort. *p*-Values comparing left- and right-sided tumors are from Pearson’s chi-square or Fisher’s exact tests for categorical variables and one-way ANOVA for continuous variables.

Variable		Left-Sided CRC (*n* = 54)	Right-Sided CRC (*n* = 7)	Total (*n* = 61)	*p*-Value
Sex	Female	24	3	27	0.937 ^a^
	Male	30	4	34	
Age (years)	Mean (SD)	44.8 (13.1)	50.7 (18.0)	45.5 (13.7)	0.289 ^b^
BMI (kg/m^2^)	Mean (SD)	22.6 (3.9)	22.0 (4.5)	22.5 (3.9)	0.832 ^b^
Family history	No	45	6	51	0.678 ^c^
	Yes	9	1	10	
Histopathology	Adenocarcinoma	46	6	52	0.933 ^a^
	Mucinous adenocarcinoma	7	1	8	
	Squamous cell carcinoma	1	0	1	
Stage	Stage I	7	2	9	0.518 ^a^
	Stage II	15	2	17	
	Stage III	32	3	35	

^a^ From Pearson’s chi-square test. ^b^ From one-way ANOVA. ^c^ From Fisher’s exact test (1-sided).

**Table 2:** Unique driver variants identified in normal colon tissue. Variants were filtered using the IntOGen COAD and READ driver gene database, resulting in 47 driver variant calls which were reduced to 26 unique variant calls across 31 patients. The “Exact Match?” column indicates whether the amino acid change observed in our cohort was identical to the variant documented in the IntOGen database at that position.

Gene	Chr	Position (GRCh37)	Ref	Alt	Documented Driver Alt	Driver Effect	Exact Match?	Patients with Variant
*APC*	5	112,173,798	C	G	A, G	Stop gained	Yes	5
	5	112,173,801	C	G	A	Stop gained	No	6
	5	112,175,162	C	A	T	Stop gained	No	1
	5	112,154,942	C	T	T	Stop gained	Yes	1
*ARID1A*	1	27,106,648	G	T	A	Missense	No	1
	1	27,106,648	G	A	A	Missense	Yes	1
*ERBB2*	17	37,879,658	G	A	A	Missense	Yes	1
	17	37,880,261	G	T	A, T	Missense	Yes	1
*FBXW7*	4	153,247,157	C	T	A	Splice donor	No	4
	4	153,271,206	G	C	C	Stop gained	Yes	2
	4	153,245,521	C	A	A	Missense	Yes	1
	4	153,247,289	G	A	A, C	Missense, non-synonymous	Yes	1
	4	153,245,494	C	T	A	Missense	No	1
*KRAS*	12	25,380,283	C	T	T	Missense	Yes	1
	12	25,398,284	C	A	A, G, T	Missense, non-synonymous	Yes	1
	12	25,398,284	C	T	A, G, T	Missense, non-synonymous	Yes	1
*PIK3CA*	3	178,936,095	A	T	C, G	Missense, non-synonymous	No	4
	3	178,952,090	G	T	C	Missense	No	1
	3	178,952,074	G	A	A, T	Missense, non-synonymous	Yes	1
*PTEN*	10	89,692,911	G	A	T	Missense	No	6
*TP53*	17	7,577,550	C	A	A, T	Missense	Yes	1
	17	7,578,388	C	T	G, T	Missense	Yes	1
	17	7,577,046	C	T	A	Stop	No	1
	17	7,578,179	C	A	A	Stop	Yes	1
	17	7,578,526	C	A	A, G, T	Missense, non-synonymous	Yes	1
	17	7,577,556	C	A	A, T	Missense	Yes	1

**Table 3:** Genes with the most significant and clinically relevant Tissue * *APC* interaction effects. Identified by FDR-adjusted ANOVA interaction *p*-value < 0.05 and at least one absolute fold change > 2. Fold changes and 95% confidence intervals represent CRC tumor tissue versus adjacent normal colon tissue in patients without (*APC* Wild) and with (*APC* Mutant) a non-synonymous potentially malignant somatic *APC* mutation detected in adjacent normal colon tissue. By the working definition of “potentially malignant”, these SMs were also detected in CRC tissues. Analysis was performed on the 46 samples with available gene expression data.

Gene	Interaction *p*-Value	*APC* Wild		*APC* Mutant	
		Fold Change	(95% CI)	Fold Change	(95% CI)
*SLC26A3*	0.015	−6.9	(−15.8 to −3.0)	−25.4	(−67.9 to −9.5)
*SELENBP1*	<0.001	−3.6	(−5.7 to −2.3)	−8.9	(−15.3 to −5.2)
*KRT20*	0.022	−2.2	(−4.0 to −1.3)	−6.2	(−12.2 to −3.1)
*CXCL14*	0.003	−1.9	(−3.0 to −1.1)	−4.3	(−7.6 to −2.4)
*MMP1*	0.045	+4.9	(+2.5 to +9.4)	+11.6	(+5.3 to +25.3)
*MMP12*	0.025	+2.6	(+1.5 to +4.6)	+7.3	(+3.7 to +14.5)
*MMP7*	0.020	+10.8	(+5.9 to +19.9)	+3.9	(+1.9 to +8.1)
*MTHFD2*	0.016	+1.5	(+1.1 to +1.9)	+2.0	(+1.4 to +2.8)
*MTHFD1L*	0.026	+2.0	(+1.6 to +2.4)	+2.3	(+1.8 to +3.0)

## Data Availability

All the supporting data are presented in the tables presented in the main manuscript and Supplementary Materials.

## Supplementary Materials

The following supporting information can be downloaded at https://www.mdpi.com/article/10.3390/cancers18183034/s1. Table S1: Mutational status of the 46 samples with available gene expression; Table S2: Genes with significant Tissue * *APC* interaction effects; Table S3: Genes with significant Tissue * *MAP3K1* interaction effects; Table S4: Genes with significant Tissue * *PIK3CA* interaction effects; Table S5: Genes with significant Tissue * *PTEN* interaction effects; Table S6: Genes with significant Tissue * *ARID1A* interaction effects; Table S7: Genes with significant Tissue * *BRCA2* interaction effects; Table S8: Genes with significant Tissue * *KMT2C* interaction effects; Table S9: Two-way ANOVA and differential expression analysis of *MTHFD1*, *MTHFD1L*, *MTHFD2*, and *MTHFD2L*; Table S10: List of abbreviations used in the manuscript.
